# The Janus face of endogenous neuronal tPA: promoting self-protection and worsening the death of neighboring neurons

**DOI:** 10.1038/s41419-024-06655-0

**Published:** 2024-04-12

**Authors:** Paul Prunotto, Pauline Marie, Laurent Lebouvier, Yannick Hommet, Denis Vivien, Carine Ali

**Affiliations:** 1 Normandie Univ, UNICAEN, INSERM, INSERM UMR-S U1237, Physiopathology and Imaging of Neurological Disorders, Institut Blood and Brain @ Caen-Normandie, Cyceron, Caen, 14000 France; 2 Department of clinical research, CHU de Caen Normandie, Caen, France

**Keywords:** Cell death in the nervous system, Stroke

## Abstract

Recombinant tissue-type plasminogen activator (r-tPA/Actilyse) stands as the prevailing pharmacological solution for treating ischemic stroke patients, of whom because their endogenous circulating tPA alone is not sufficient to rescue reperfusion and to promote favorable outcome. Beyond the tPA contributed by circulating endothelial cells and hepatocytes, neurons also express tPA, sparking debates regarding its impact on neuronal fate ranging from pro-survival to neurotoxic properties. In order to investigate the role of neuronal tPA during brain injuries, we developed models leading to its conditional deletion in neurons, employing AAV9-pPlat-GFP and AAV9-pPlat-Cre-GFP along with tPA floxed mice. These models were subjected to N-methyl-D-aspartate (NMDA)-induced excitotoxicity or thromboembolic ischemic stroke in mice. Initially, we established that our AAV9 constructs selectively transduce neurons, bypassing other brain cell types. Subsequently, we demonstrated that tPA-expressing neurons exhibit greater resistance against NMDA-induced excitotoxicity compared to tPA negative neurons. The targeted removal of tPA in neurons heightened the susceptibility of these neurons to cell death and prevented a paracrine neurotoxic effect on tPA non-expressing neurons. Under ischemic conditions, the self-neuroprotective influence of tPA encompassed both excitatory (GFP^+^/Tbr1^+^) and inhibitory (GFP^+^/GABA^+^) neurons. Our data indicate that endogenous neuronal tPA is a protective or deleterious factor against neuronal death in an excitotoxic/ischemic context, depending on whether it acts as an autocrine or a paracrine mediator.

## Background

Recombinant tissue-type plasminogen activator (r-tPA) (or its derivate Tenecteplase) is the only pharmacological treatment at the acute phase of ischemic stroke, based on its ability to convert plasminogen into active plasmin and thus to promote thrombolysis [1]. tPA is also a multifaceted mediator of brain physiopathology [2–4]. In the brain parenchyma, tPA is expressed in different structures such as the hippocampus, amygdala or somatosensory cortex and by different cell types including activated microglia, oligodendrocytes and neurons [5–7] Regarding neurons, tPA has been identified in vitro, in neuronal cell lines and in primary cultured neurons [8, 9]. In cultured cortical and hippocampal neurons, tPA is stored in endosomal and exosomal vesicles in axons and dendrites [10–12]. After activity-dependent exocytosis, tPA released in the synaptic cleft can either potentiate N-methyl-D-Aspartate (NMDA) receptor-dependent glutamatergic signaling, or be recycled in astrocytes, thus behaving as a neuromodulator [12–15]. The expression of tPA by neurons in vivo has been shown in hippocampal CA1 neurons (and corresponding mossy fibers) and in cortical neurons, although in these latter, immunodetection was not possible with conventional techniques [6, 16, 17].

The potential neuronal toxicity of exogenous tPA is well documented, in particular under excitotoxic conditions [14, 18], although protection against apoptosis [19] and/or autophagy [20] have also been reported. By contrast, the actual role of endogenous tPA in the control of neuronal fate, especially during stroke remains highly debated. A meta-analysis of the impact of tPA deficiency in mechanical stroke models [21] concluded that endogenous tPA has no effect on brain infarction, due to the huge heterogeneity of results (i.e., both smaller and bigger lesions reported). This likely arises from different extents of contribution of circulating *versus* parenchymal tPA to stroke pathogenic events, depending on the model used [22–24]. The use of tPA KO mice has yielded a plethora of data regarding the global toxic or protective impact of tPA. However, this mouse model does not allow elucidating the implications of tPA at a cellular scale. To our knowledge, Tsirka’s team is the only one that has specifically investigated cell-type specific contribution of tPA, by re-introducing tPA expression in microglia or in neurons in tPA deficient mice [25]. However, in this study, the expression of tPA was under the control of the macrophage colony-stimulating factor (M-CSF) proto-oncogene promotor or the neurofilament light chain (NF-L) promoter, leading to an over-expression of the protease, even in cells that do not normally express it.

Instead, here we used serotype 9 adeno-associated viral (AAV) constructs with the endogenous tPA promotor (pPlat). We used two original constructs (AAV9-pPlat-GFP and AAV9-pPlat-Cre-GFP) to unmask or delete endogenous tPA expression only in transduced neurons, in mice expressing lox-P sites in exon-3 of the Plat gene. Our aim was to investigate the in vivo influence of endogenous tPA specifically released by neurons at the early stages of excitotoxic and ischemic conditions. We demonstrate that tPA is expressed by some GABAergic and glutamatergic cortical neurons, and this neuronal tPA displays a self-protective role against NMDA-mediated excitotoxicity and ischemic neuronal death. Simultaneously, neuronal tPA contributes to the excitotoxic death of neighboring tPA non-expressing neurons.

## Results

### tPA is expressed by excitatory and inhibitory cortical neurons in vivo

**Fig. 1:**
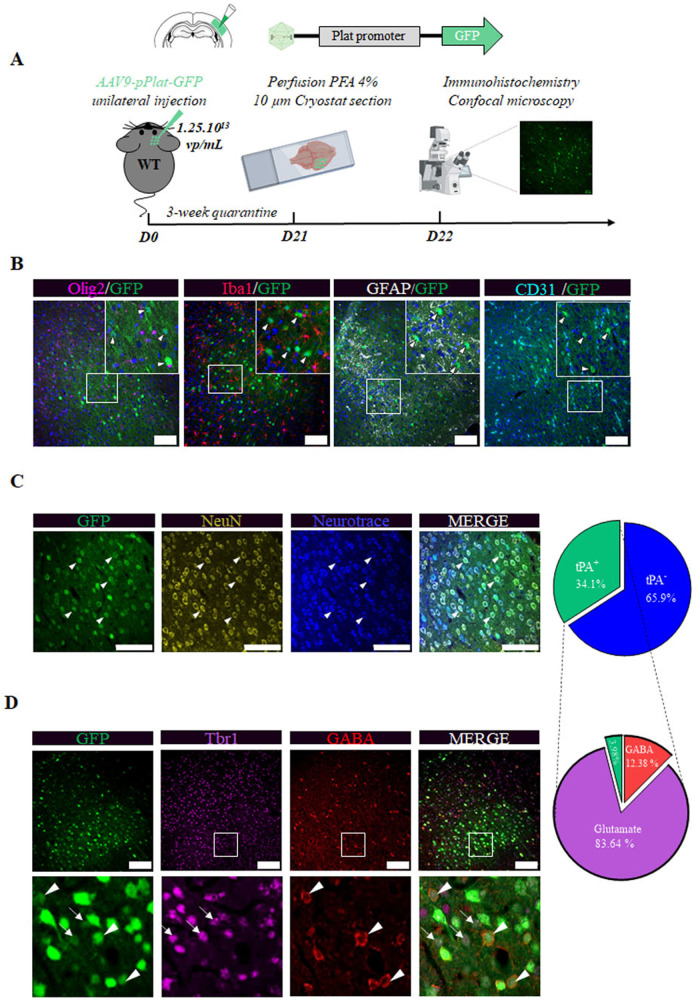
tPA is expressed by cortical neurons *in vivo.* **A** Schematic experimental protocol showing the unilateral injection of 2 × 0.25 µL of 1.25 × 10^13^ vp/mL of AAV9-pPlat-GFP in tPA flox^−/−^ mice (WT, -0.3 mm AP, ±3.3 mm ML, –0.8 & 0.4 mm DV). **B** Representative images of immunohistochemical analyses showing that AAV9-pPlat-GFP does not transduce oligodendrocytes (magenta), microglia (red), astrocytes (gray), and blood vessels (cyan). Scale bar = 100 µm, ×20. **C** Representative images and corresponding quantifications of immunohistochemical analyses showing that AAV9-Plat -GFP transduces around one third of neurons (yellow; NeuN and Blue; Neurotrace). Scale bar = 100 µm, objective = ×40. **D** Quantification of the proportion of neurons GABA-expressing (a marker of inhibitory neurons) or tbr1 (a marker of excitatory neurons) among the population of GFP (tPA) positive neurons, in the transduced area. A white dashed arrow indicates the colocalization between the TBR1 marker and GFP, whereas an isolated white triangle denotes the colocalization between the GABA marker and GFP. Scale bar=100 µm, ×20.

We used an AAV9-pPlat-GFP construct, allowing GFP to serve as a reporter (ie., GFP positive cells are tPA-expressing cells, while GFP negative cells are tPA-non-expressing cells). Three weeks after the intracortical injection of AAV9-pPlat-GFP viral particles, the brains of swiss mice were collected for immunohistochemical analyses (Fig. 1A). Neither microglia (Iba1 marker, red), oligodendrocytes (Olig2 marker, magenta), astrocytes (GFAP marker, white) nor endothelial cells (CD31 marker, cyan) expressed GFP (white arrows) (Fig. 1B). By contrast, GFP was associated with the neuronal marker NeuN (yellow) or the Neurotrace Nissl staining (blue) (Fig. 1C). These data show that in our experiments, our constructs only transduce neurons. Around 34% of all cells expressing NeuN and stained by the Neurotrace Nissl staining were positive for GFP (Fig. 1C), showing that one third of neurons in this cortical area express tPA. Around 84% of GFP positive neurons co-expressed the transcription factor tbr1, a marker of excitatory neurons (magenta) while 12% expressed GABA, a marker of inhibitory interneurons (red) (Fig. 1D). tPA is thus expressed by inhibitory and excitatory cortical neurons.

### In the cortex, tPA-expressing neurons are more resistant to excitotoxicity than tPA non-expressing neurons

**Fig. 2:**
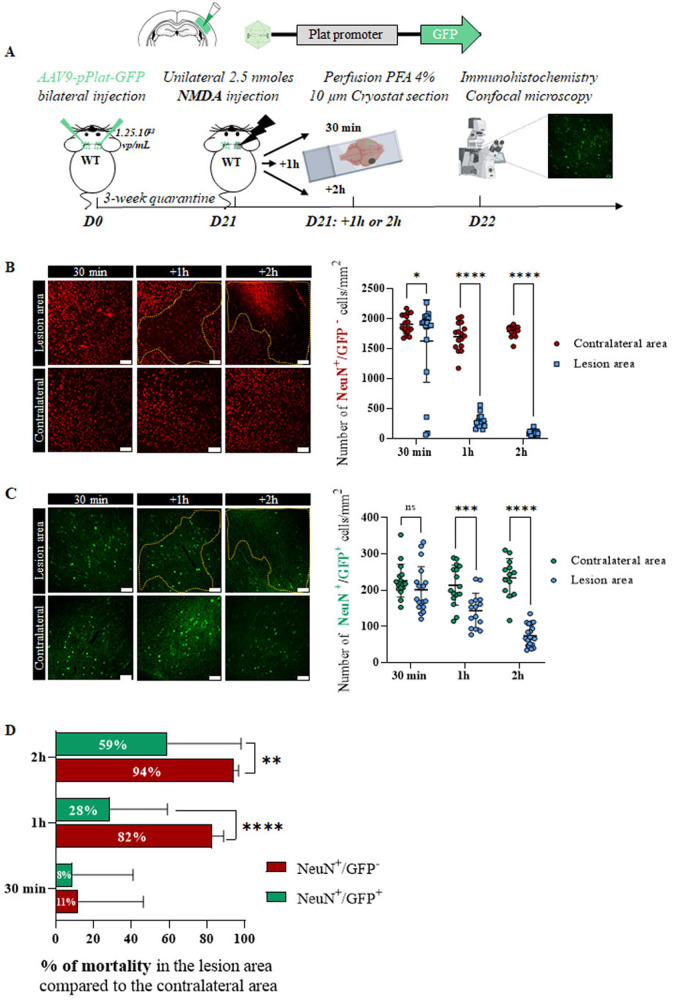
tPA cortical neurons are more resistant to excitotoxicity than non tPA neurons. **A** Schematic experimental protocol showing the AAV9-pPlat-GFP injection in both somatosensory cortices of swiss mice, followed by the unilateral injection of NMDA into the right somatosensory cortex. Brain were collected at 30 min, 1 and 2 h after the NMDA injection to immunohistochemical analyses. **B**, **C** Representative images and quantification of the number of tPA non-expressing neurons (red) and tPA-expressing neurons (green) in the lesion area compared to the contralateral area, at 30 min, 1 and 2 h post NMDA injection (*N* = 4 mice for 30 min and 1 h groups, *N* = 5 mice for 2 h group; Two-way ANOVA, multiple comparison, Bonferroni post-hoc; *p* < 0.05 = *, *p* < 0.001 = ***, *p* < 0.0001 = ****). Scale bar = 100 µm, ×20. **D**. Quantification of the percentage of tPA non-expressing (red) and tPA-expressing neurons (green) mortality at 30 min, 1 and 2 h post NMDA injection (*N* = 4 mice for 30 min and 1 h groups, *N* = 5 mice for 2 h group; two-way ANOVA, Multiple Comparison, Bonferroni post-hoc; *p* < 0.01 = **, *p* < 0.0001 = ****).

With the aim of elucidating the role of neuronal tPA in excitotoxic phenomena, we injected AAV9-pPlat-GFP into both the right and left somatosensory cortices of mice, and 3 weeks after, excitotoxicity was induced by a stereotaxic injection of the NMDA (2.5 nmoles) in the right somatosensory cortex. Brains were then collected at three acute time points after injury: 30 min, 1 h, and 2 h post NMDA injection (Fig. 2A). Following NMDA injection, the density of tPA non-expressing cortical neurons was significantly reduced in the lesion as early as 30 min, while that of tPA-expressing neurons did not reduce before 1 h (Fig. 2B, C). Accordingly, the mortality of tPA non-expressing cortical neurons increased with time to reach 94% mortality at 2 h, while that of tPA expressing was 59% at the same time (Fig. 2D).

### The Janus face of the neuronal tPA: self-protective for tPA-expressing neurons but neurotoxic for tPA negative neighboring neurons at the acute phase of excitotoxicity

**Fig. 3:**
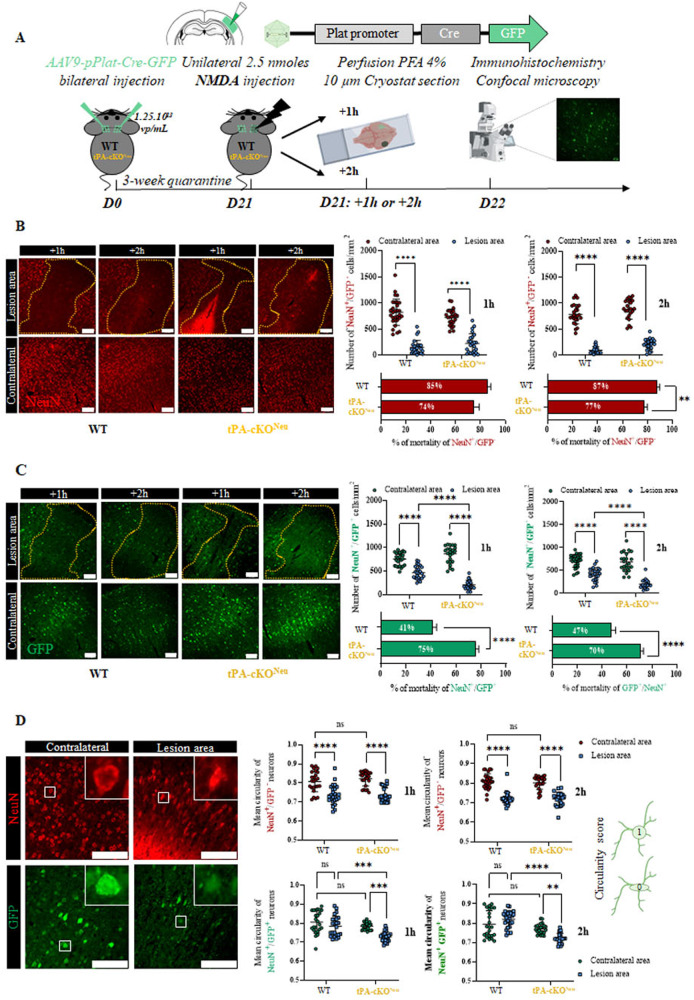
tPA from cortical neurons promotes neuronal survival at the acute phase of excitotoxicity. **A** Schematic experimental protocol showing first, the AAV9-Plat-Cre-GFP injection in tPA ^−/−^ (WT) or flox^+/+^ (tPA-cKO^Neu^) mice and second, the NMDA injection followed by IHC. **B**, **C** Representative images and quantification of the number of tPA non-expressing neurons (red) and tPA-expressing neurons (green) in the lesion area compared to the contralateral area, at 1 and 2 h post NMDA injection (*N* = 5 (+1 h) and *N* = 6 (+2 h) mice per time for WT and *N* = 4 (+1 h) and 4 (+2 h) for tPA-cKO^Neu^; Two-way ANOVA, Multiple comparison, Bonferroni post hoc; *p* < 0.0001 = ****). The percentage of mortality was calculated for both genotypes at 1 and 2 h post excitotoxicity induction (*N* = 5 (+1 h) and *N* = 6 (+2 h) mice per time for WT and *N* = 4 (+1 h) and 4 (+2 h) for tPA-cKO^Neu^. Mann–Whitney test; *p* < 0.01 = ** for NeuN^+^/GFP^−^ cells and Unpaired *t*-test, *p* < 0.0001 = **** for NeuN^+^/GFP^+^ cells. Scale bar = 100 µm, ×20. **D** Representative images and quantification of the tPA non-expressing (red) and tPA-expressing (green) cortical neuronal circularity in the lesion area and the contralateral area of WT or tPA-cKO^Neu^ mice, 1 and 2 h post excitotoxicity induction (*N* = 5 (+1 h) and *N* = 6 (+2 h) mice per time for WT and *N* = 4 (+1 h) and 4 (+2 h) for tPA-cKO^Neu^; Two-way ANOVA, Multiple comparison, Bonferroni post hoc; *p* < 0.01 = **, *p* < 0.001 = ***, *p* < 0.0001 = ****). Scale bar = 100 µm, ×40.

We then investigated whether endogenous neuronal tPA participates in the acute phase of excitotoxic neuronal death. tPA Flox^−/−^ or tPA Flox^+/+^ mice were bilaterally injected with AAV9-pPlat-Cre-GFP into the somatosensory cortex, allowing either unmasking (Flox^−/−^: wild-type, WT) or unmasking and deleting (tPA Flox^+/+^: tPA conditional knockout in neurons, tPA-cKO^Neu^) neuronal tPA in transduced neurons. After 3 weeks, mice received a unilateral injection of NMDA (2.5 nmoles) into the right somatosensory cortex and brain tissues were collected for immunohistochemical analyses at 1 h or 2 h post NMDA injection (Fig. 3A).

The mortality of tPA non-expressing neurons (NeuN^+^/GFP^−^, red) was severely increased in the lesion area compared to the contralateral area and being more severe in WT mice at 2 h (85% for WT mice and 74% for tPA-cKO^Neu^ mice at 1-h and 87% for WT mice and 77% for tPA-cKO^Neu^ mice at 2 h, Fig. 3B). Again, we observed that tPA-expressing neurons (NeuN^+^/GFP^+^) are more resistant to excitotoxicity than tPA non-expressing neurons (41% *versus* 85% mortality at 1 h and 47 *versus* 87% mortality at 2 h, Fig. 3B, C). Strikingly, the mortality of tPA-expressing neurons was significantly higher in tPA-cKO^Neu^ mice compared to WT mice (75 versus 41% at 1 h and 70 versus 47% at 2 h, Fig. 3C). Accordingly, the circularity of tPA non-expressing neurons (NeuN^+^/GFP^–^ cells), an index of cell suffering [26], was equally reduced in both genotypes at 1 and 2-h after NMDA injection. By contrast, circularity of tPA-expressing neurons was not affected by NMDA injection in WT mice but was reduced in tPA-cKO^Neu^ mice (Fig. 3D).

These data evidence a self-protective effect of tPA on tPA-expressing neurons against NMDA-induced excitotoxicity, and by contrast a deleterious effect of neuronal tPA for neighboring tPA non-expressing neurons.

### Neuronal tPA acts as a self-neuroprotective mediator at the acute phase of ischemic stroke

**Fig. 4:**
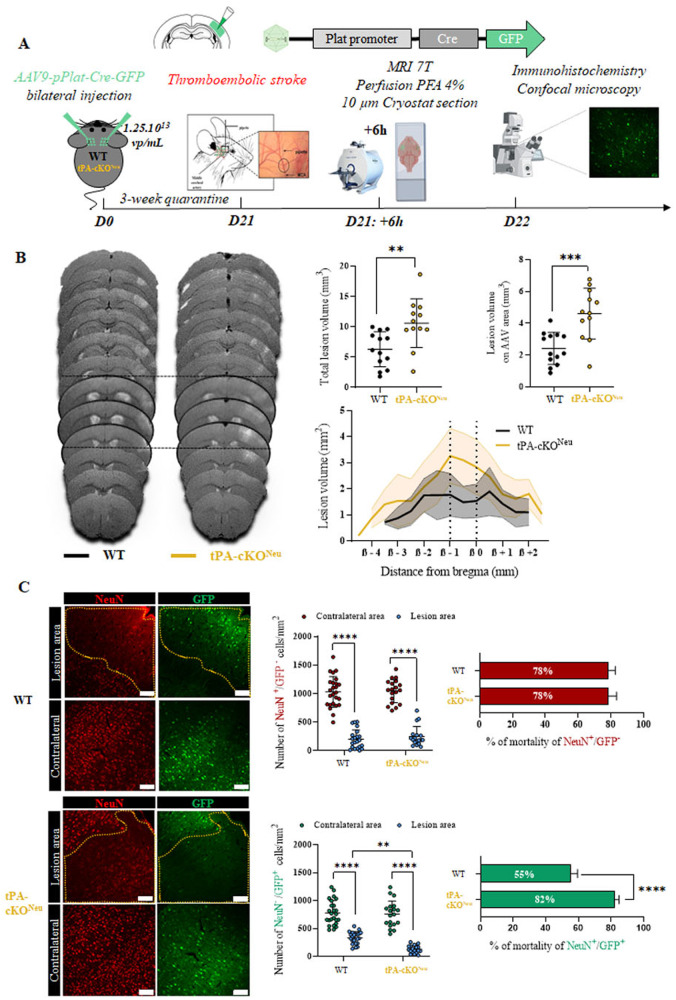
Neuronal tPA acts as a neuroprotective mediator at the acute phase of ischemic stroke. **A** Schematic experimental protocol showing the AAV9-Plat-Cre-GFP injection, the induction of the thromboembolic model of stroke, the MRI T2 sequences and the IHC performed 6 h after stroke. **B** Illustration Representative images and quantification of the total lesion volume and the lesion volume in the AAV area injection for WT and tPA-cKO^Neu^ mice (*N* = 13 for WT and *N* = 12 for tPA-cKO^Neu^; Unpaired *t*-test; *p* < 0.01= **, *p* < 0.001 = ***). **C** Representative images and quantification of tPA non-expressing neurons (red) and tPA-expressing neurons (green) in the lesion area compared to the contralateral area, 6-h post ischemia (*N* = 5 WT and *N* = 4 tPA-cKO^Neu^; Two-way ANOVA, Multiple comparison, Bonferroni post hoc; *p* < 0.01 = **, *p* < 0.0001 = ****. The percentage of mortality was calculated for both genotypes at 6-h post ischemia (*N* = 5 for WT and *N* = 6 for tPA-cKO^Neu^ mice. Mann–Whitney test for NeuN^+^/GFP^-^ cells and Unpaired *t*-test, *p* < 0.0001 = **** for NeuN^+^/GFP^+^ cells. Scale bar = 100 µm, ×20.

The role of endogenous neuronal tPA at the acute phase of ischemic stroke was then investigated in WT or tPA-cKO^Neu^ mice subjected to a model of thromboembolic occlusion of middle cerebral artery occlusion (MCAO). Subsequently, ischemic lesions were assessed 6 h post occlusion using T2 and T2* MRI sequences and immunohistochemical analyses (Fig. 4A). Interestingly, we found that both genotypes have a similar hypoperfusion in the lesion area (Supplementary fig. 2). tPA-cKO^Neu^ mice exhibited larger lesion volumes than WT mice, when considering both the total lesion volume (6.2 mm^3^ for WT and 10.5 mm^3^ for tPA-cKO^Neu^) and the lesion volume within the anatomic slices covering the AAV9 injection sites (2.4 mm^3^ for WT and 4.5 mm^3^ for tPA-cKO^Neu^, Fig. 4B). The density of tPA non-expressing neurons was similarly reduced in both genotypes. Consistent with this, the mortality was equal in WT and tPA-cKO^Neu^ mice (78%, Fig. 4C). Regarding tPA-expressing neurons, their density and survival was more severe in tPA-cKO^Neu^ mice compared to WT mice (82 versus 55% mortality in tPA-cKO^Neu^ and WT mice, respectively, Fig. 4C).

These data evidence that during thromboembolic stroke, neuronal tPA reduces infarct lesions, induces a self-protective effect on tPA-expressing neurons, but has no effect on neighboring tPA non-expressing neurons.

### Excitatory and inhibitory tPA-expressing neurons have a similar resistance to stroke

**Fig. 5:**
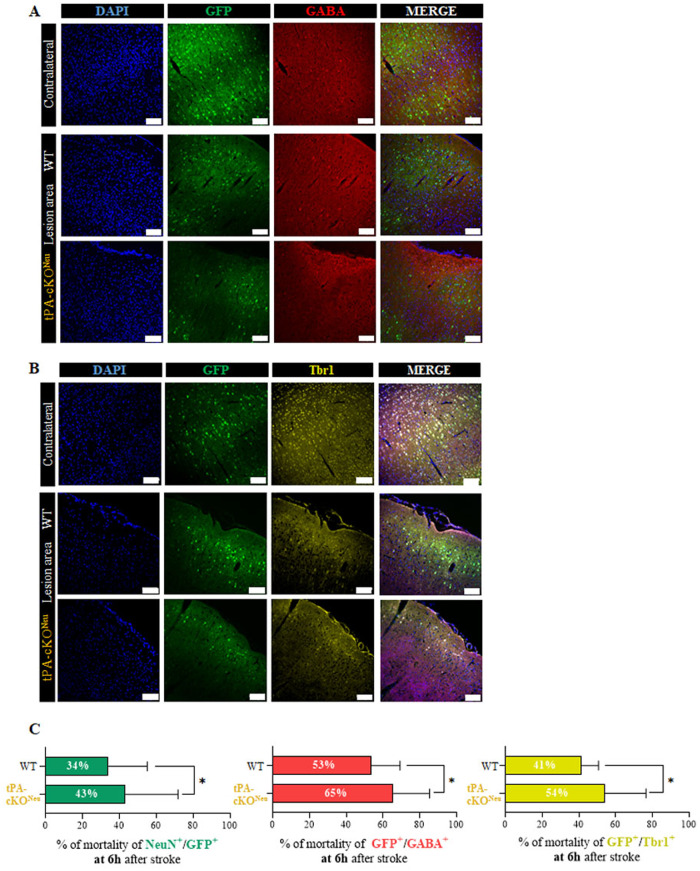
Excitatory and inhibitory tPA-expressing neurons have a similar sensitivity to excitotoxicity. **A**, **B** Representative images and quantification (**C**) of the percentage of tPA-expressing neurons (green), inhibitory tPA-expressing neurons (GABA^+^, red) and tPA-expressing neurons (red) and excitatory tPA-expressing neurons (tbr1^+^, yellow) mortality 6-h post ischemia for WT and tPA-cKO^Neu^ mice (*N* = 5 for WT and 4 for tPA-cKO^Neu^ mice; unpaired *t*-test; *p* < 0.05 = *). Scale bar = 100 µm, ×20.

Finally, we investigated the acute vulnerability of the different subtypes of tPA-expressing neurons to stroke. First, we confirmed that WT mice had a lower proportion of tPA positive neuronal loss compared to tPA-cKO^Neu^ mice, with a mortality of 15% and 53%, respectively (Fig. 5A). The loss of tPA-expressing inhibitory neurons (GFP^+^/GABA^+^ cells) was lower in WT (42% mortality) than in tPA-cKO^Neu^ (64% mortality) mice (Fig. 5A, B). Similarly, the loss of tPA positive glutamatergic neurons, was significantly lower in WT compare to tPA-cKO^Neu^ mice (54% versus 37% loss, Fig. 5C, E).

## Discussion

Our data (illustrated on Supplementary Fig. 3) indicate that endogenous tPA is expressed by several subtypes of cortical neurons and that this neuronal tPA acts as a protective or deleterious factor against neuronal death in an excitotoxic/ischemic context, depending on whether it acts as an autocrine factor or a paracrine one.

Whether endogenous tPA is protective or neurotoxic is still a subject of debate [14, 27, 28]. To date, most previous conclusions were drawn from studies performed at later (acute/subacute) stages of disease models, and did not allow distinguishing the effect of tPA depending on its cellular source. Here we demonstrate for the first time that a significant proportion of excitatory and inhibitory neurons express tPA and that neuronal tPA, at physiological levels, confers neuroprotection at hyper acute stages against excitotoxicity and stroke. We have also highlighted that the ambivalence of neuronal tPA, being protective for tPA-expressing neurons (autocrine effect) but neurotoxic for neurons that do not express it (paracrine effect).

Many previous studies have used tPA knockout (tPA KO) mice to investigate the roles of tPA, but it has been subsequently demonstrated that this model has some limitations [29]. Indeed, tPA KO mice carry a specific chromosomal segment derived from the 129 strain, which co-segregates with the targeted Plat allele. Moreover, alterations in additional genes such as *Arhgef18*, *Mcf2l*, and *Mcph1* can introduce complexities when interpreting data [4, 30]. Also, tPA KO mice do not allow studying the contribution of different compartments (circulation versus parenchyma, [24]) or of a specific cell population in the brain parenchyma.

Some authors have used inhibitors of tPA (serpins), as an alternative to tPA KO mice. Such approaches have the advantage to locally and acutely block tPA [24]. However, it is important to keep in mind that serpins block tPA catalytic activity, but unlikely the “cytokine-like” effects of tPA [5, 7, 31]. Moreover, serpin/tPA complexes may conserve some biological activities, and/or accelerate the clearance of tPA [32]. Of note, Rogove and Tsirka (1998) could highlight the role of microglia-derived tPA in excitotoxicity, by infusing macrophage/microglial inhibiting factor (MIF) to block microglial activation [33]. The same group also used genetic approaches to reintroduce tPA expression only in either neurons or microglial cells in tPA KO mice [25]. They could thus provide convincing evidence that tPA exerts cell-type specific functions under excitotoxic conditions.

Adding to the previous literature, here, we used a new mouse strain, with floxed alleles flanking exon 3 of the tPA gene [4, 34]. This targeted genetic modification differs from tPA KO mice, as it allows creating conditional tPA KO. Furthermore, it is known that the immunodetection of tPA in neurons is very challenging using conventional methods [6]. Accordingly, we have also used two adeno-viral constructs, allowing in vivo detecting (AAV9-pPlat-GFP) or deleting and detecting tPA (AAV9-pPlat-Cre-GFP) specifically in neurons, thanks to the specificity of the AAV9 serotype in our conditions [35, 36].

In our hands, investigating early stages of pathogenic pathways, tPA-expressing cortical neurons are protected from excitotoxicity and stroke compared to tPA non-expressing neurons, and this protection is due to their own production of tPA. We also observed that tPA non-expressing neurons exhibit a higher mortality in WT mice compared to tPA-cKO^Neu^ mice, after excitotoxicity, but not after stroke. Neuronal tPA may thus have early opposite autocrine and paracrine activities during excitotoxicity, and only early autocrine activities during stroke.

Despite their above-mentioned limits, tPA KO have shed light on the multifaceted aspects and modes of action of tPA. Tsirka and collaborators first demonstrated that tPA KO mice exhibited resistance to excitotoxic neuronal death induced by kainate injection in the hippocampus [27, 37, 38]. In cultured cortical neurons, endogenous tPA did not alter kainate or α-amino-3-hydroxy-5-methyl-4-isoxazolepropionic acid (AMPA)-induced neuronal death, but aggravated NMDA-induced calcium influx and neuronal death. This was shown by using tPA KO neuronal cultures, or addition of its natural inhibitors, plasminogen activator inhibitor type 1 (PAI-1, [14]) or neuroserpin [39]. Furthermore, in vitro, activated microglial cells can release tPA and promote apoptotic cell death of hippocampal neurons [40]. Centonze and colleagues have also suggested that tPA may enhance ischemia-induced delayed neuronal apoptosis, based on findings that striatal slices from tPA KO mice subjected to oxygen and glucose deprivation (OGD) are unable to display NMDA-dependent post-ischemic long-term synaptic potentiation [41]. However, other studies have shown neuroprotective effects of endogenous tPA. Echeverry and collaborators revealed that tPA is a neuroprotective mediator of ischemic preconditioning in the hippocampus [42], which was later suggested to be driven by a plasmin and NMDA receptors-dependent neuronal expression of TNF, and the subsequent upregulation of cyclin-dependent kinase inhibitor p21, an anti-apoptotic factor [43]. This group has also shown that endogenous tPA promotes cell survival in cortical neurons exposed to OGD by recruiting the mTOR pathway and HIF-1α [44]. Additionally, tPA induces the expression of the glucose transporter GLUT3, facilitating glucose uptake by cortical neurons and reducing lesion volumes following intra-striatal NMDA injection [45], thanks to NMDA receptors containing the GluN2A subunit, rapid phosphorylation of extracellular signal-regulated kinases (ERK), activation of CREB, and induction of the neuroprotective transcription factor Atf3. The authors also demonstrated that OGD leads to tPA release into the synaptic cleft, activating AMPK and enhancing neuronal glucose recovery and ATP production, a process dependent on NMDA receptors. More recently, Lemarchand and collaborators demonstrated that hippocampal slices from tPA KO mice were more susceptible to OGD compared to WT slices, an effect dependent on EGF receptors [46]. Recently, it has been shown that tPA exerted neuroprotective effects by increasing AMPK phosphorylation and FUNDC1 expression, a protein involved in autophagy, as demonstrated in a model of 1-h occlusion of the middle cerebral artery. In this model, tPA KO mice exhibited increased neuronal death compared to their wild-type littermates [47].

In conclusion, our study provides compelling evidence for the impact of endogenous neuronal tPA, at physiological levels, on neuronal fate during the early phases of excitotoxicity and stroke. The use of our innovative AAV9 constructs allowed to uncover the self-neuroprotective role of tPA derived from tPA-expressing neurons, in the context of excitotoxicity or ischemic stroke. Here, we focused on the somatosensory cortex, which is histologically and functionally affected by the anatomical level of vessel occlusion in our model. It would be interesting to use our construct to investigate whether our findings extend to other cortical or brain regions in which tPA expressing and tPA non-expressing neurons may co-exist.

We also highlight the potential toxic paracrine effect of tPA from tPA-expressing on tPA non-expressing neurons in a context of excitotoxicity.

These findings contribute to our understanding of the intricate roles of tPA in neuronal function and highlight its potential as a therapeutic target for ischemic stroke treatment.

## Materials and methods

### Animals

Experiments were conducted in compliance with the French ethical law (Decree 2013-118) and the European Communities Council guidelines (2010/63/EU). The protocols were approved by the local ethics committee, under the French Ministry of Research and Higher Education (agreement numbers Cenomexa #25267 and #37835 and Ce5/2012/062), and all appropriate international, national, and institutional guidelines for the care and the use of animals were followed. All in vivo or ex vivo studies were performed on 5 to 8-week-old male Swiss mice (30-35 g) or C57BL6J mice (tPA Flox^+/+^: tPA-cKO^Neu^ mice when injected by the AAV9-pPlat-Cre-GFP and their control tPA Flox^−/−^ littermates: WT mice, 20–25 g). The animals were kept under a 12-h light/12-h dark cycle with free access to food and water in standard polypropylene cages (22 × 37 × 19 cm, Charles River, L’Arbresle, France; 3 mice per cage).

The randomization of animals into groups was achieved through the utilization of artificial intelligence (AI). This was employed to ensure a truly random allocation, enhancing the robustness of the experimental design.

Animals were initially excluded from the study if comments on their surgeries were unfavorable, or if the pain threshold was exceeded during and after the surgeries. If the NMDA induced lesion or stroke lesion occurred in a different region than AAV injection, the animals were excluded from the study. This inclusion/exclusion criterion was rigorously enforced to maintain the quality and experimental consistency of the collected data.

### Adeno-associated virus (AAV) production

Viral particles were provided by Gilles Bonvento and Alexis Bemelmans (INSERM U1169/MIRCen CEA, Fontenay aux Roses 92265, France). The pPlat-GFP clone, which encodes for the green fluorescent protein under the control of a 1.2-kb sequence of the human Plasminogen Activator promoter and the pPlat-Cre-GFP clone, which encodes for the Cre recombinase and the GFP under the control of a 1.2-kb sequence of the human Plasminogen Activator promoter, were purchased from GeneCopoeia (Catalog No.: HPRM12655-PF02). The cDNA encoding for the Cre recombinase was subcloned by fusion into the pPlat-GFP (In-fusion HD cloning Kit Clontech-Takarabio). The pPlat-GFP and the pPlat-Cre-GFP were then subcloned in the pDONR221 for AAV production. The constructs were amplified in Escherichia coli JM109 cells and purified by a Nucleobond endotoxin-free plasmid DNA PC 2000 kit (Macherey-Nagel) according to the manufacturer’s instructions. The AAV9 serotype vector was used in this study. Self-complementary AAV vectors expressing the Plat-Cre-GFP construct were produced by transfecting HEK293 cells with the adenovirus helper plasmid (pXX6-80), the AAV packaging plasmid carrying the rep2 and cap8 genes, and the AAV2 shuttle plasmid containing the Plat-Cre-GFP transgene in a self-complementary genome. The recombinant vectors were purified by ultracentrifugation on a discontinuous iodixanol density gradient and then dialyzed against the formulation buffer of the vector stocks (0.5 mmol/l MgCl_2_ and 1.25 mmol/l KCl in phosphate-buffered saline (PBSMK)) with five buffer changes of 3 h each. The physical particles were quantified by real-time PCR, and the vector titers were expressed as viral genomes per milliliter (vg/mL).

### Intracortical stereotaxic injection of AAV9

For AAV injections, 5-week-old mice were deeply anesthetized under 5% isoflurane in a gas mixture consisting of 70% nitrous oxide and 30% oxygen. The depth of anesthesia was monitored by the absence of reflexes upon paw pinch. The mice were then placed in a stereotaxic frame (Harvard Apparatus), under anesthesia (2.5% isoflurane; 70% N_2_O/30%O_2_) with the aid of ear bars to immobilize the cranial box. Local lidocaine was applied to prevent pain caused by ear bars. The animal temperature was maintained at 37 °C using a heating pad placed under the animal and monitored by a rectal probe. The skull was cleaned with alcohol and then with Betadine to limit infections during surgery and at the time of suture. The skull was incised, and the bregma coordinates were recorded, a craniotomy was performed at the injection site using a dental drill, allowing then inserting the AAV-containing micropipette at the chosen stereotaxic coordinates. The coordinates used for intracortical injections in the primary somatosensory cortex were −0.5 mm AP (anteroposterior), ±3.3 mm ML (medial/lateral), and −0.8 and 0.4-mm D (depth from the brain surface). AAV9 was injected in both primary somatosensory cortices. The viral particles (1.25 × 10^13^ vg/mL; 2 × 0.25 μL per hemisphere) were injected at a rate of 0.2 μL/min to allow progressive diffusion in the brain parenchyma and to preserve the tissue. The micropipette was left in place for 3 min after the complete injection volume had been administered and then slowly withdrawn to prevent the reflux of viral particles from the injection site. The surgical site was cleaned with Betadine and the skin sutured. The duration of anesthesia was identical for all experimental groups (30 min). Three weeks after infections, animals were subjected to either direct immunohistochemistry, NMDA injection or stroke. This procedure has already been used and validated, showing that AAV9-pPlat-Cre-GFP does indeed remove tPA from mossy fibers of hippocampal neurons in tPA Flox^+/+^ mice, without deleting but demasking it in tPA Flox ^−/−^ [4].

### Intracortical NMDA injection

For the unilateral injections of NMDA, 8-week-old mice were deeply anesthetized under 5% isoflurane in a gas mixture consisting in 70% nitrous oxide and 30% oxygen. The depth of anesthesia was monitored by the absence of reflexes upon paw pinch. The mice were then placed in a stereotaxic frame (Harvard Apparatus), under anesthesia (2.5% isoflurane; 70% N_2_O/30% O_2_) with the aid of ear bars to immobilize the cranial box. Local lidocaine was applied to prevent pain caused by ear bars. The animal temperature was maintained at 37 °C using a heating pad placed under the animal and monitored by a rectal probe. The skull was cleaned with alcohol and then with Betadine to limit infections during surgery and at the time of suture. The skull was incised, and the bregma coordinates were recorded, a craniotomy was performed at the injection site using a dental drill, allowing then inserting the NMDA-containing micropipette at the chosen stereotaxic coordinates. The coordinates used for intracortical injections in the right primary somatosensory cortex were −0.5 mm AP (anteroposterior), +3.3 mm ML (medial/lateral), and −0.6 D (depth from the brain surface). NMDA (2.5 nanomoles; 1 × 0.33 μL) were injected at a rate of 0.2 μL/min to allow progressive diffusion in the brain parenchyma and to preserve the tissue. The micropipette was left in place for 3 min after the complete injection volume had been administered and then slowly withdrawn to prevent the reflux of NMDA solution from the injection site. At 30 min, 1 h, or 2 h after NMDA injection, animals were euthanized for immunohistochemical analysis.

### Thromboembolic model of stroke

Eight-week-old mice were deeply anesthetized under 5% isoflurane in a gas mixture consisting of 70% nitrous oxide and 30% oxygen. The depth of anesthesia was verified by the absence of reflexes when the paw was pinched. The mice were then placed in a stereotaxic frame using ear bars under anesthesia (2.5% isoflurane; 60% N_2_O/40% O_2_) to immobilize the skull. Local lidocaine application was performed to prevent pain caused by the ear bars. The animal temperature was maintained at 37 °C using a warming pad placed under the animal, and its temperature was monitored using a rectal probe. An incision was made between the right eye and ear, and the masseter muscle was dissociated from the lateral aspect of the parietal bone and then cut into two parts. After locating the middle cerebral artery (MCA), a craniotomy was performed at the M1-M2 segments of the bifurcation of the MCA. The skull was cooled and cleaned with saline during the craniotomy. The dura mater was then excised. Cerebral ischemia was induced by in situ injection of murine thrombin (1 μL, 1 IU, Kordia) using a hematologic micropipette fixed to the articulated arm of the stereotaxic frame where the animal was positioned. The duration of anesthesia was the same for all animals: approximately 15 min before occlusion and 25 min after, totaling 40 min. After protecting the craniotomy with a hemostatic compress (Pangen®), the skin was sutured. Animals were euthanized after MRI session, 6 h post ischemia.

### Magnetic resonance imaging

Six hours after stroke onset, lesion volumes, hemorrhagic transformations (HT) and tissular perfusion were measured using a Pharmascan 7-Tesla/12 cm Magnetic Resonance Imaging (7T-MRI, Bruker). The mice were deeply anesthetized with 5% isoflurane and maintained with 1.5–2% isoflurane in 30% O_2_/70% N_2_O during image acquisition. T2-weighted images were used to analyze lesion sizes, while T2*-weighted sequences were used to detect hemorrhagic events. Tissular perfusion were acquired by using an Arterial spin labeling (ASL) sequence. Lesion sizes were quantified using ImageJ software. The duration of the MRI examination was estimated to be 25 min, including mouse placement in the cradle and computer sequence preparation.

### Antibodies

The following antibodies and/or tracers were used: mouse anti-NeuN (1:800, MAB377, Merck); rabbit anti-Green fluorescent protein (GFP, 1:1000, ab6556, abcam); goat anti-Green fluorescent protein (GFP, 1:1000, ab5450, abcam); chicken anti-Glial Fibrillary Acidic Protein (GFAP, 1:2000, ab4674, abcam); rabbit anti-Ionized calcium binding adapter molecule 1 (Iba-1, 1:1000, 019-19741, Fujifilm); rabbit anti- Gamma aminobutyric acid (GABA, 1:500, A2052, Sigma Aldrich); Rabbit anti-CD31 (1:1000, ab28364, abcam); NeuroTrace™ 435/455 Blue Fluorescent Nissl Stain (1:300, N21479, Invitrogen); rabbit anti- Oligodendrocyte transcription factor (Olig2, 1:3000, ab9610, Millipore); chicken anti-T-box brain transcription factor 1 (tbr1, 1:250, ab2261, Merck).

### Immunohistochemistry

Mice were deeply anesthetized with a mixture of 5% isoflurane in 70% nitrous oxide and 30% oxygen. A transcardial perfusion was performed using 0.9% NaCl solution with 3% heparin, followed by fixation with a solution containing 4% paraformaldehyde in 0.1 M PBS with a pH of 7.4. The brains were removed, cryoprotected for 48 h in a 20% sucrose solution in 0.1 M PBS (pH 7.4), and then frozen in Tissue-Tek. Cryostat sections of 10 µm were collected on Poly-Lysine slides and stored at −80 °C. Primary antibodies were incubated with the sections overnight at room temperature. Corresponding Fab'2-conjugated secondary antibodies were diluted at 1:800 (Jackson Immunoresearch). Images were captured using a Leica DM6000 microscope with a CoolSnap camera and visualized with Metamorph 7.0 software (Molecular Devices) or Leica TCS SP8 Confocal/STED 3× microscope with an oil-immersion 40×, 1.44-N.A. objective at a resolution of 1024 × 1024 pixels, a speed of 600 Hz, and a step size of 35 µm.

### Histological analysis

Images were further processed using ImageJ software or QuPath Open Software. All analyses were performed blindly to the genotype. The experimenter was blinded to the genotype but not to the type of AAV injected during AAV administration. Additionally, for surgeries related to NMDA injection and stroke modeling, the experimenter remained blind to the genotype of the subjects. For the quantification of neuronal loss in tPA-expressing neurons or tPA-non-expressing neurons, the number of NeuN^+^/GFP^−^ or NeuN^+^/GFP^+^ cells in the lesion area or in the contralateral area were manually counted (at least 5 images per hemisphere, from 4 mice per group for 30 min and 1-h group and 5 mice for 2 h group). For the quantification of neuronal loss in tPA-cKO^Neu^ or WT mice after NMDA injection or stroke, the number of DAPI^+^, NeuN^+^ or Neurotrace^+^ cells overlapping or not with GFP^+^ cells in the lesion area or in the contralateral area were manually counted (at least 5 images per hemisphere, from at least 4 mice per group).

The presence of the Cre recombinase in the transduces neurons was controlled by immunohistochemistry, as seen in supplementary fig. 1 (anti-mouse; MAB3120).

Neuronal circularity was calculated using the following equation: circularity = $4\pi x{\frac{{\rm{area}^{{2}}}}{{\rm{perimetre}}}}$ [26]. This score provides an indication of morphological changes in neurons, with a score close to 1 indicating a normal neuron and a score close to 0 indicating a suffering neuron.

### Statistics

All results are presented as means ± SD. Statistical analyses were performed using GraphPad Prism software (version 9.0). Prior to initiating the experiments, we employed the G Power test to determine the requisite sample size for detecting differences between our experimental conditions, thereby adhering to the principles of the 3Rs and minimizing the number of animals utilized. In some cases, the analysis of the datasets using parametric approaches proved to be inadequate due to the violation of normality assumptions, as indicated by the results of Shapiro-Wilk tests. Hence, appropriate selection of either parametric or non-parametric approaches was made as per the requirement. We conducted two-way ANOVA tests with multiple comparisons to analyze the number of tPA-non-expressing or tPA-expressing neurons in the lesion or the contralateral area and the absolute number of neurons (NeuN) or tPA-expressing neurons (NeuN^+^/GFP^+^) in the lesion and contralateral cortical areas between WT and tPA-cKO^Neu^ mice at 1-h and 2-h post NMDA injection. The same test was used to quantify the mortality of neurons after stroke. The inclusion of multiple comparisons allowed us to examine the specific differences between groups and time points, providing a comprehensive understanding of the effects of genotype and time on neuronal populations in these regions. *Post-hoc* tests using the Bonferroni correction were performed to determine significant differences between specific groups. Same analyses were performed for the experimental condition 6 h post induction of ischemic stroke. The statistical test used to compare genotypes for lesion volumes was the parametric Student’s *t*-test. For the other comparisons, we conducted two-way ANOVA tests with multiple comparisons, followed by Bonferroni *post-hoc* tests. These analyses allowed us to assess significant differences between groups while considering the effects of multiple factors simultaneously. All tests were two-tailed.

### Supplementary information

supplementary materials

## Data Availability

The data that support the findings of this study are available on request from the corresponding author.
