# Supplementary material for: The Janus face of endogenous neuronal tPA: promoting self-protection and worsening the death of neighboring neurons
Source: Cell Death Dis. 2024 Apr 12;15(4):261. doi: 10.1038/s41419-024-06655-0 (PMC11014960; doi:10.1038/s41419-024-06655-0)
Supplement: Supplementary file 1 — supplementary materials [file 41419_2024_6655_MOESM1_ESM.pdf]

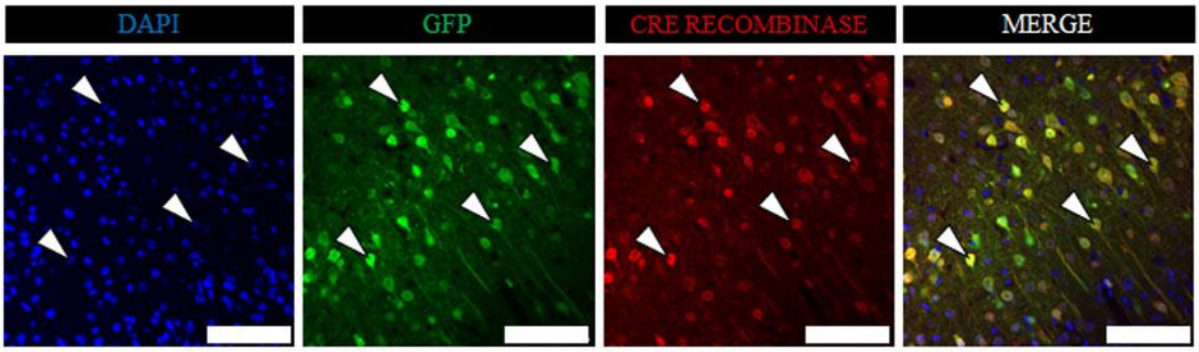

Supplementary Figure 1

Control immunohistochemistry against the Cre-recombinase and the GFP. Scale bar=100µm, X40.

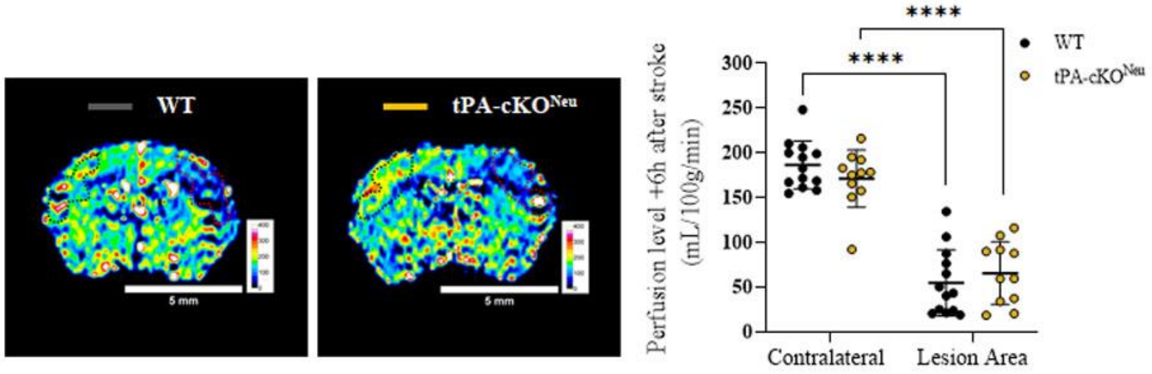

Supplementary Figure 2

Representative images and quantification of ASLMRI sequencing at 6-hours after stroke, in the contralateral and the lesion area of WT and tPA-cKO^{Neu} mice (N=13 for WT mice and N=11 for tPA-cKO^{Neu} mice).

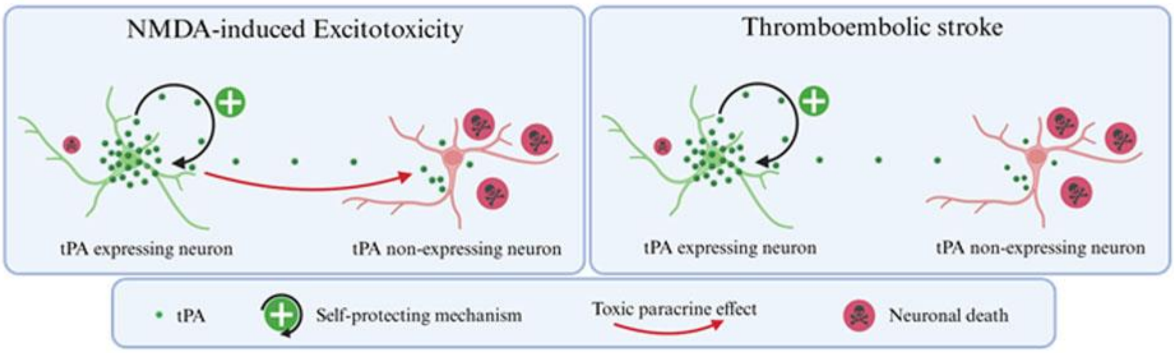

<u>Supplementary Figure 3</u>

Schematic representation of the (autocrine) self-protective action of neuronal tPA together with its potential (paracrine) impact on tPA non-expressing neurons after excitotoxic or ischemic conditions.
